# Immobile Tricuspid Valve: Incidental Finding in a Case of Terminal Cardiomyopathy Due to Thalassemia Major

**DOI:** 10.5935/abc.20190195

**Published:** 2019-09

**Authors:** Erman Cilsal

**Affiliations:** Adana City Education and Research Hospital, Adana - Turkey

**Keywords:** Cardiomyopathies, beta-Thalassemia/genetics, delta-Thalassemia/genetics, Arritmias Cardíaca, Tricuspid Valve/abnormalities, Echocardiography/methods

## Introduction

Thalassemia Major is an inherited disorder caused by impaired synthesis of the B globin chain and characterized by ineffective erythropoiesis that requires regular, lifelong transfusion therapy, which creates a state of iron overload.^1^ Once reticuloendothelial stores saturate, iron deposition increases in myocardium such as other parenchymal tissues.^2^ Cardiac complications due to this deposition are the leading cause of death. After a silent first decade, iron deposits in the cardiac tissue lead to arrhythmias, systolic and diastolic dysfunction, and congestive heart failure in the second or third decade.^3^ In this case report, we present an adolescent girl who did not receive regular iron chelation therapy and had cardiomyopathy, arrhythmia and immobile tricuspid valve secondary to thalassemia major.

### Case presentation

A 14-year-old Syrian girl with Thalassemia Major presented to the emergency room with a three-month history of increasing fatigue, dyspnea, and abdominal distension. Her medical history revealed that she had been diagnosed with Thalassemia Major at the age of one year old, and she received irregular erythrocyte transfusion and iron chelation therapy in her country. It was learned that the compliance for previous blood transfusion and chelation therapy was very poor. On general examination, she was undernourished with short stature (body weight < 25 p, height < 3p) and the physical examination revealed dyspnea with a typical facial thalassemic feature without cyanosis.

**Figure 1:**
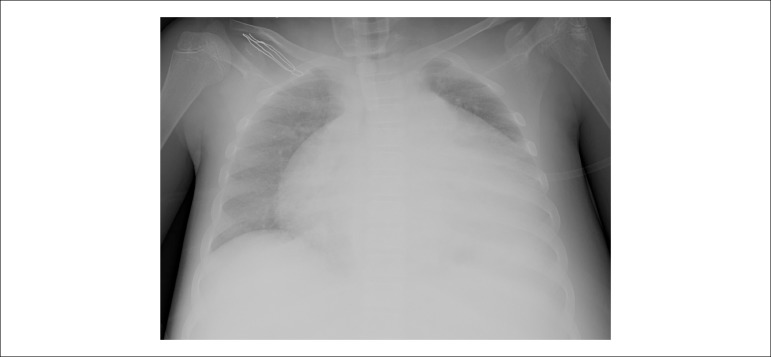
Chest X-Ray of the patient.

**Figure 2:**
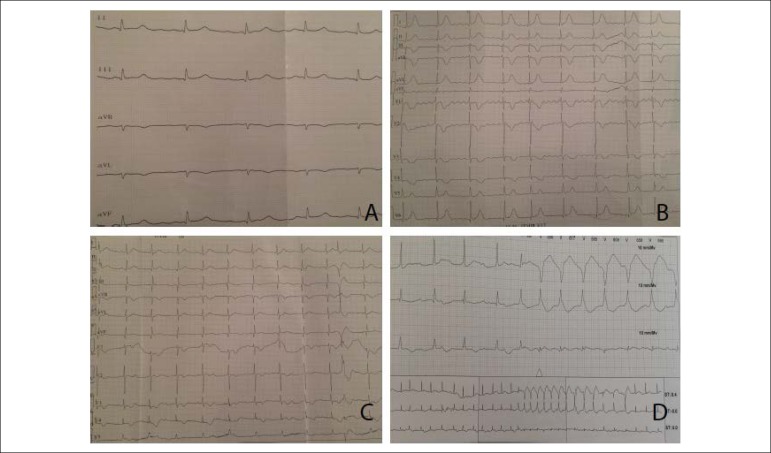
Electrocardiography of the patient. (A). Sinus rhythm with QTc prolongation (B). Atrial flutter (C). Atrioventricular dissociation and ventricular extra-systole (D). Holter monitorization revealed non-sustained ventricular tachycardia.

**Figure 3:**
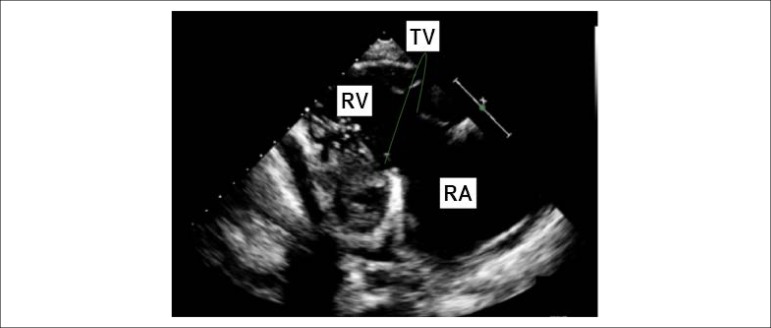
Transthoracic echocardiography from the parasternal view by tilting transducer inferomedially exploring the right atrium (RA) and right ventricle (RV) inflow tract; immobile leaflets of the tricuspid valve (TV) leading to severe insufficiency.

Chest x-ray showed areas of consolidation on both sides of the lungs and increased cardiothoracic ratio (Figure 1). The electrocardiogram showed sinus rhythm with 70/min heart rate and prolongation of QTc value with 0.46 seconds (Figure 2-A). Transthoracic echocardiography revealed both ventricle systolic and diastolic ventricular dysfunction, left ventricle ejection fraction was 48% and fractional shortening was 24% were calculated with a mild left ventricle dilatation (Table 1). Mild-moderate mitral regurgitation and trivial pericardial effusion were also observed. Right ventricular inflow view in systole showing thickened, immobile leaflets of tricuspid valve in a fixed open position, causing mal-coaptation and severe regurgitation without stenosis (see Figure 3 and Video 1). Apical four-chamber view in diastole showed immobile leaflets of tricuspid valve in a fixed open position, as showed by the color Doppler (Video 2) (See additional files Video 3, 4 and 5). Right atrial, right ventricle dilatation and minimal pulmonary regurgitation with mild pulmonary hypertension were also observed.

**Table 1:** Echocardiographic measurements of the patient

Data	Values
**M-Mode Measurements**	
LVID, cm	4.9
Ejection Fraction	48
Fractional shortening, %	24
RVID, cm	4.8
**Doppler Measurements**	
Tricuspid E, cm/s	81
Tricuspid A, cm/s	25
Tricuspid E/A	3.2
**Tissue Doppler Measurements (RV)**	
E' cm/s	12.1
A' cm/s	7.8
E'/A'	1.55
E/E'	6.7
S'	11.4
IVCT, ms	65
IVRT, ms	78
RV MPI	62

A: peak late diastolic velocity; A': late diastolic velocity; E: peak early diastolic velocity; E': early diastolic velocity; ET: ejection time; IVCT: isovolumic contraction time; IVRT: isovolumic relation time; LVIDd: Left ventricular internal diastolic diameter; MPI: myocardial performance index; RV: right ventricle; RVID: right ventricular internal diameter; S': systolic velocity; Tissue Doppler imaging of the tricuspid valve.

**Video 1:**
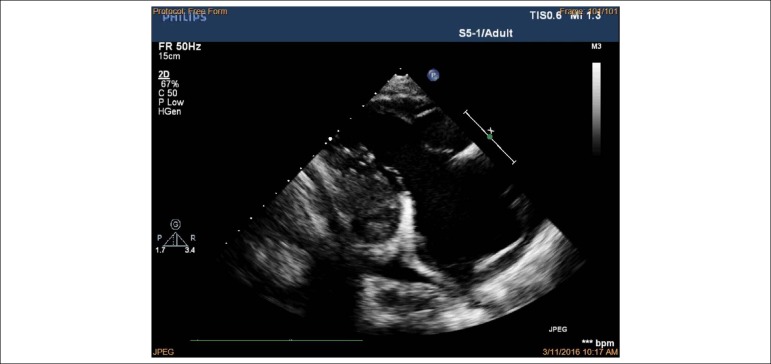
Transthoracic echocardiography from the right ventricular inflow view in systole showing immobile leaflets of tricuspid valve in a fixed open position, causing mal-coaptation and severe regurgitation. To view the video click on the link: https://bit.ly/2lTc6iX

**Video 2:**
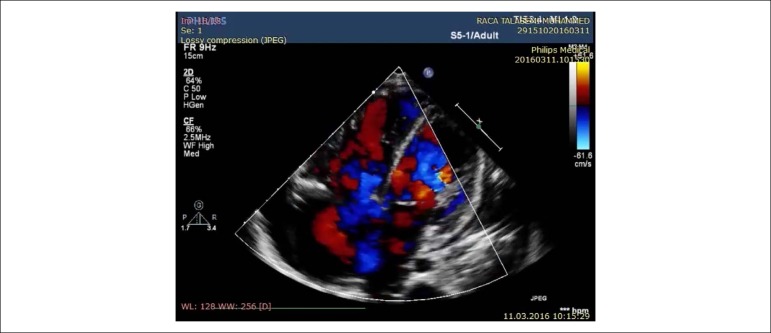
Transthoracic echocardiography from the apical four-chamber by color Doppler view showed immobile leaflets of tricuspid valve in a fixed open position. To view the video click on the link: https://bit.ly/2lTc6iX

**Video 3:**
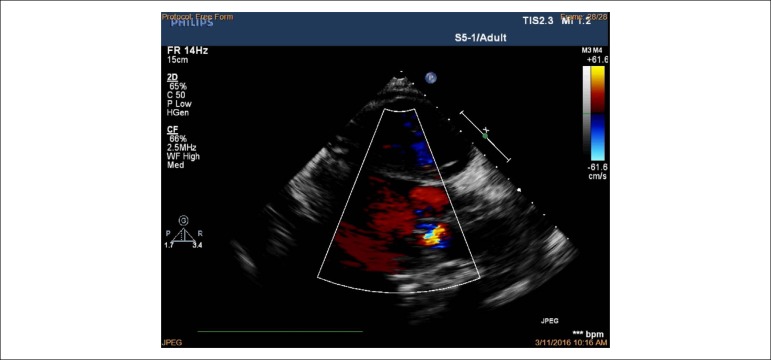
Transthoracic echocardiography from the parasternal long axis view with color Doppler showing mild mitral regurgitation. To view the video click on the link: https://bit.ly/2lTc6iX

**Video 4:**
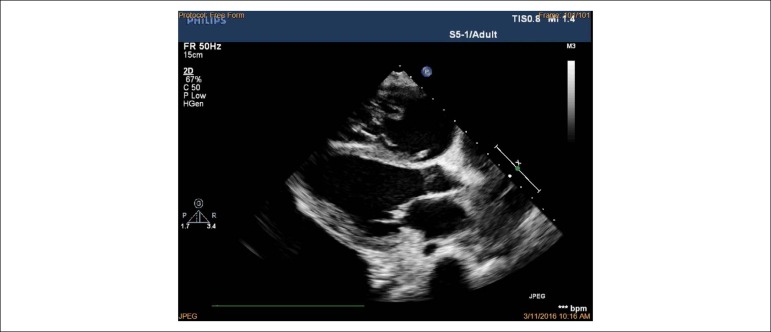
Transthoracic echocardiography from the parasternal long axis view showing normal systolic function of the left ventricle and minimal pericardial effusion. To view the video click on the link: https://bit.ly/2lTc6iX

**Video 5:**
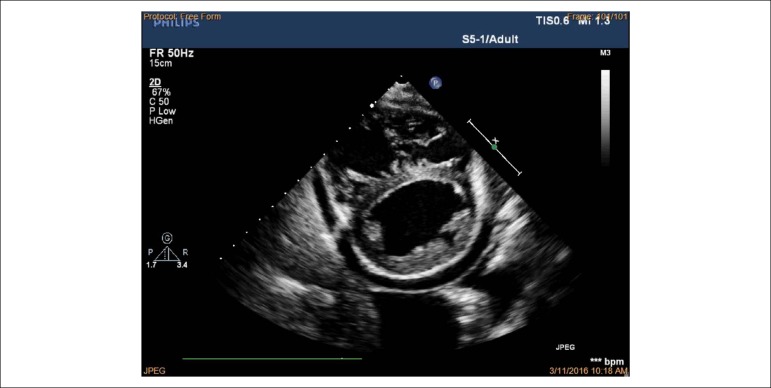
Transthoracic echocardiography from the parastrenal short axis view showed enlargement of the right ventricle and pericardial effusion. To view the video click on the link: https://bit.ly/2lTc6iX

After hospitalization in the intensive care unit, inotropes, diuretics and iron chelation treatment (Dopamine, Dobutamine, Furosemide infusion, Propranolol, Enalapril, Aldactone and Deferoxamine, Deferiprone therapy) started as soon as possible. Cardiac enzymes were sent to screen possible myocarditis, and D-dimer was sent to detect pulmonary thromboembolism. Results were found to be negative. On the seventh day of hospitalization, the electrocardiogram showed atrial flutter (Figure 2-B). Therefore, digoxin and low molecular weight heparin treatment were also started. In the second week, the patient developed acute renal insufficiency and the electrocardiogram showed atrioventricular dissociation and ventricular extra-systole (Figure 2-C). Immediately after the digoxin treatment had been stopped, amiodarone infusion has started. Holter monitorization revealed atrioventricular dissociation and non-sustained ventricular tachycardia (Figure 2-D). Blood level of Digoxin was within normal reference values. Serial echocardiography was performed and no difference has been observed in the cardiac parameters during the hospitalization. Despite atrioventricular dissociation, we decided to follow-up her without pacemaker implantation due to hemodynamic stability. Although secondary prevention of implantable cardioverter defibrillator was decided after the patient was taken to control ventricular arrhythmias with amiodarone, she died due to ventricular tachycardia on the 22nd day of hospitalization.

## Discussion

In thalassemia, cardiovascular system involvement is pivotal in the prognosis and quality of life. Iron overload cardiomyopathy is the leading cause of mortality accounts up to 67% and 71% in thalassemia.^4^ As iron overload, multiple factors such as chronic anemia, hypersplenism, non-progressive restrictive lung disease also lead to cardiac complications in Thalassemia Major.^5^ Iron is mainly stored in myocytes and other cells in the form of free iron, also ferritin and hemosiderin. Free iron, which is referred to as labile cellular iron, is the most toxic form of iron and also the most accessible form for chelation. The goal of iron chelation therapy is to reduce the iron deposition especially in plasma and other tissues. In some cases, these heart complications were reported as reversible with early detection of iron overload and response to regular iron chelation therapy.^6^

Cardiac magnetic resonance imaging (MRI) is the gold standard for detecting myocardial iron deposition. In our case, cardiac MRI was not performed due to lack of experienced staff in our hospital. Progressive increase of brain natriuretic peptide assay is highly sensitive and specific in the diagnosis of heart failure. In our patient, brain natriuretic peptide levels were markedly elevated.

Conventional standard echocardiography exhibits pathologic findings at advanced stages of cardiac involvement. The assessment of the ventricular function involves two different phenotypes. The first one is ‘dilated cardiomyopathy’ phenotype revealed by with left-right ventricular dilatation and reduced contractility, which cause congestive heart failure. The second one is ‘restrictive cardiomyopathy’ phenotype revealed by restrictive left-right ventricular filling resulting in pulmonary hypertension, right ventricular dilatation, and heart failure.^7^

In this report, our patient had impaired cardiac functions similar to both dilated and restrictive patterns of cardiomyopathy. Right, and left ventricle contractility was reduced which led to congestive heart failure. Both ventricle diastolic dimensions were increased. Assessment with pulsed and pulsed tissue Doppler demonstrated left and right ventricle diastolic dysfunction. Factors that may cause pulmonary hypertension in patients with thalassemia include elevated pulmonary resistance due to high volume of blood flow, elevated shear forces, hypercoagulable state secondary to splenectomy and nitric oxide formation after chronic hemolysis. Although right heart failure may develop secondary to pulmonary hypertension, in thalassemic patients, it may also develop in the absence of elevated pulmonary hypertension.^8^

In our case, typical stenotic changes and doming that seen in rheumatic diseases were not present in the tricuspid leaflet. Uniformly, mildly thickened tricuspid leaflets were present with a relatively fixed valve orifice without stenosis. Cardiac carcinoid usually affects the right cardiac chamber of the heart and results in a similar presentation. However, it is not reported in the pediatric age group in the literature. Nevertheless, carcinoid tumor should also be considered as a differential diagnosis in isolated advanced tricuspid valve involvement.^9^ In our case in contrast to the carcinoid tumor, the tricuspid valve did not exhibit very bright echoes secondary to fibrous plaques that are deposited on the endocardium of the leaflets.^10^ Biogenic amine levels in plasma and urine samples were found to be in the normal range that excluded diagnosis of carcinoid tumor. The related literature indicates similar findings in patients with thalassemia; however, illustration of echocardiograms in children is not satisfactory. Aessopos et al.^6^ reported valvular involvement including leaflet thickening (48%), endocardial calcification (20%), and left-sided valve regurgitation in adult patients with thalassemia intermedia.^6^ In our case, the patient had serious dysrhythmias due to endocardial involvement, contraction and relaxation dysfunction due to myocardial involvement, and severe leaflet disorder due to valvular involvement. We herein report an extraordinary thalassemia major patient with immobile and non-stenotic tricuspid valve that emerges as a part of the terminal phase of the cardiomyopathy.

## Conclusion

Thalassemia major patients, especially those who do not receive regular chelation therapy, are under great risk of cardiac involvement. Early detection and regular treatment regimen enhance their survival and quality of life. We firstly present an immobile tricuspid valve in an adolescent girl. This very rare case of severe cardiac findings due to iron deposition is associated with endocardial, myocardial and valvular involvement. In patients with thalassemia, these end-stage complications of the cardiovascular system are irreversible despite treatment.
